# Personal

**Published:** 1916-06

**Authors:** 


### PERSONAL.

---

**Announcement of removal, travel, and other matters of interest are requested. Please report errors in the listing of any physician in the State and other directories, that we may co-operate with the State Society in securing a correct list.**

---

Dr. Harry R. Trick of Buffalo announces his removal to 522 W. Ferry Street, and his attention to General Surgery.

---

Dr. Herbert A. Smith of Buffalo announces his removal to 566 Delaware Avenue.

---

Dr. Nelson W. Strohm of Buffalo announces his removal to 566 Delaware Avenue.

---

Dr. A. H. Aaron announces the opening of offices at Suite 5, 494 Franklin Street, Buffalo, and his limitation to Diseases of the Digestive Organs.

---

Dr. Lee Masten Francis of Buffalo, announces the removal of his office to 636 Delaware Avenue.

---

Dr. Arthur C. Eisbein has been appointed Diagnostician of the Buffalo Health Dept. at a salary of $2,000.

---

Dr. Charles S. Butler of Buffalo, announces his retirement from dental practice. He has been appointed Field Secretary in charge of the fund for superannuated and needy clergymen of the Presbyterian Church.

---

Dr. Ernest L. Vogenau and Dr. Julius Y. Cohen had the misfortune of knocking down pedestrians with their automobiles, recently, the latter accident resulting fatally. No blame is attached to the drivers.

---

Dr. Bendis has been appointed interne at the J. N. Adam Memorial Hospital, Perrysburg, at a salary of $600 and maintenance.

---

Dr. W. S. Renner of Buffalo visited White Sulphur Springs, W. Va., early in May.

---

Dr. Prescott Le Breton attended the meeting of the American Orthopaedic Assn. at Washington, in May.

---

Dr. G. H. Peddle has been elected treasurer of the Perry Rifle Club.

---

Dr. Harry C. Dumville has resigned as bacteriologist of Niagara Falls, after a service of four years. Miss Hazel Robinson will succeed him.

---

Dr. John G. Stowe of Buffalo announces the removal of his office to 566 Delaware Avenue.

---

Dr. F. A. Mendlein has been elected Vice-President of the North Jefferson Street (Buffalo) Business Men's Assn.

---

The following Buffalo physicians have passed the civil service examination for the position of city physician: Herbert H. Bauckus, Peter J. Barone, Charles Leone, Francis H. Lennan, Leon S. Kurek, Mansfield G. Levy, Wm. E. Dieffenbach, John H. Wild, Wilfred H. Baynes. The following have been nominated for appointment, by the mayor: John H. Wild, Charles Leone, Leon S. Kurek, Herbert H. Bauckus.

